# Supplementary material for: Diagnostic Considerations of Intermetatarsal Bursitis: A Systematic Review
Source: Diagnostics (Basel). 2023 Jan 6;13(2):211. doi: 10.3390/diagnostics13020211 (PMC9857655; doi:10.3390/diagnostics13020211)
Supplement: Supplementary file 1 [file diagnostics-13-00211-s001.zip › diagnostics-2115789-supplementary.pdf]

Supplementary Table S1 - Extended search history

**PubMed search September 2022:**<br>"Morton Neuroma"[Mesh] OR ((intermetatarsal neuritis) OR (intermetatarsal neuralgia) OR (intermetatarsal neuroma) OR (forefoot neuroma) OR (interdigital neuroma)) OR ((morton* neuroma) OR (morton* neuralgia) OR (morton* metatarsalgia) OR (civinini* neuroma) OR (civinini* metatarsalgia)) OR ((intermetatars* OR interdigital OR forefoot) AND (bursitis OR bursa))<br><br>*Result: 757 hits*	**Embase search September 2022:**<br>1. Exp Morton neuroma/<br>2. (morton* adj3 (neurom* or neuralgia or metatarsalgia)).tw,kw.<br>3. (civinini* adj3 (neurom* or metatarsalgia)).tw,kw.<br>4. ((intermetatarsal or forefoot or interdigital) adj3 (neurom* or neuralgia or neurit*)).tw,kw.<br>5. ((intermetatars* or forefoot or interdigital) adj3 (bursitis or bursa*)).tw,kw.<br>6. 1 or 2 or 3 or 4 or 5<br><br>*Result: 843 hits*
**Cochrane Library search September 2022:**<br>1. MeSH descriptor: [Morton Neuroma] explode all trees<br>2. (morton* near/3 (neurom* OR neuralgia OR metatarsalgia)).ti,ab,kw<br>3. ((intermetatarsal or forefoot or interdigital) near/3 (neurom* or neuralgia or neurit*)). ti, ab,kw<br>4. ((intermetatars* OR forefoot OR interdigital) near/3 (bursitis or bursa*)).ti, ab,kw<br>5. #1 OR #2 OR #3 OR #4<br><br>*Results: 70 hits*	**Web of Science search September 2022:**<br>1. TS=(morton* AND (neurom* OR neuralgia OR metatarsalgia))<br>2. TS=((intermetatarsal OR forefoot OR interdigital) near/3 (neurom* or neuralgia or neurit*))<br>3. TS=((intermetatars* OR forefoot OR interdigital) near/3 (bursitis or bursa*))<br>4. TS=(civinini* near/3 (neurom* or metatarsalgia))<br>5. #1 OR #2 OR #3 OR #4<br><br>*Results: 641hits*

Supplementary Table S2 - Results from each study regarding intermetatarsal bursitis

Author and year	Results regarding the intermetatarsal bursa
S1.1 Anatomical studies	
Bossley et al. 1980 [1]	The intermetatarsal bursa in the second and third interspace distending distal and past the transverse metatarsal ligament to meet the neurovascular bundle. In the fourth interspace, the bursa ends at the distal border of the transverse metatarsal ligament.
Chauveaux et al. 1987 [5]	The intermetatarsal bursa was found to be in close relation with the digital plantar nerves, especially in the 2nd and 3rd intermetatarsal spaces where the bursas are placed very anteriorly. The authors describe that any increase in size of the bursa will produce compression of the collateral nerves.
Theumann et al. 2001 [15]	MR bursography with anatomic correlation showed the detailed anatomy of the intermetatarsal bursa. In the 2nd and 3rd intermetatarsal space the bursa extend past the distal border of the deep transverse metatarsal ligament and becomes intermetatarsophalangeal. Here the bursa comes in close contact (1 mm) of the neurovascular bundle. The bursas could not be identified on MRI before contrast injection in any of the cases.
S1.2 Studies of patients with metatarsalgia or Morton's neuroma	
Albano et al. 2021 [16]	IMB was found in 81% of the feet (n=27) pre-treatment, which was reduced to 25% three months post-treatment.
Awerbuch et al. 1982 [17]	Treatment with intrabursal corticosteroid injection was successful in 28/50 pts. The remaining 22 pts proceeded to surgery. A subtotal excision of the intermetatarsal bursa and the digital nerve was performed in 20 pts, of which 17 had complete relief. In two pts, who had a neurectomy, experienced temporary relief. 20/50 pts had rheumatoid disease at initial presentation or developed it later. Swelling over the painful cleft was found in 45/50 pts.
Bencardino et al. 2000 [18]	Distended intermetatarsal bursae were found in two pts with symptomatic MN and one pt with clinically silent MN. Prevalence of clinically silent MN was 33%.
Bossley et al. 1980 [1]	D: Radiography with contrast injected in the bursa did not show any difference in appearance between symptomatic and asymptomatic pts. T: 11 pts with typical MN had an injection of steroid into the bursa. 1 year later four of the 11 pts remained symptom free.
Cohen et al. 2016 [11]	A significant larger hypoechoic mass was found at the preoperative US, compared to the US images of the specimen. Thickened or scarred bursal tissue was found around the nerve and interdigital vessels at surgery in all cases. The authors conclude that "neuroma-bursal complex" is a more fitting description for the condition.
Espinosa et al. 2010 [10]	IMB was found in 6/22 (27%) in the symptomatic group and in 6/68 (9%) in the asymptomatic group. Histologic examination of surgery specimens found digital nerve with surrounding bursitis in all cases.
Hassouna et al. 2007 [14]	In 31% of the 45 pts IMB was diagnosed at US evaluation. 39 of the 45 pts participated in the treatment study; 28% experienced complete pain relief after treatment.
Iagnocco et al. 2001 [13]	IMB was found in 20.5% of the pts. For subgroups of pts clinically suspected MN (n=14) and pts with RA (n=13), 21.4% and 23.1% had IMB on US, respectively.
Umans et al. 2014 [19]	10/40 (25%) pts with plantar plate tear, had IMB in the adjacent interspace. 13/56 (23%) pts without plantar plate tear, had IMB. Five in the second interspace and eight in the third.
Volpe et al. 1998 [12]	According to the micro- and macroscopic findings, surgical specimens were divided into grades from 0-3. Five pts had grade 1 changes where the interdigital nerve and intermetatarsal bursa formed a mass. Surgical results from this group were varying.
Zanetti et al. 1997 [20]	Fluid was visible in minimum one intermetatarsal bursa in 47/70 participants (67%). Prevalence relative to each intermetatarsal space was 20%, 47%, 49% and 0% for the first to the fourth interspace. Correlation between fluid in interspace and presence of MN findings was significant for the 3rd interspace but not the 2nd.
S1.3 Studies of patients with autoimmune disorders	
Albtoush et al. 2019 [21]	13 cases of IMB were found in the six pts. Five pts had unilateral involvement and one had bilateral. IMB was localized in the first (n=4), second (n=4) and third (n=5).
Bowen et al. 2010 [22]	At baseline, 83.3% (50/60) of the feet had US detectable FFB hypertrophy, at 12-weeks it was 75% (39/52). This change was not significant, but the pt reported disability had significantly improved.<br>*FFB defined as both intermetatarsal bursa and plantar (adventitious) bursa.*

Bowen et al. 2010 [23]	At 1-year follow-up 120/149 participants attended: 93.3% had min. one US detectable FFB. 90.8% had visible intermetatarsal bursae, most frequently found in the fourth interspace followed by first, third and second.<br>Changes in FFB at US was significantly correlated with changes in patient-reported foot impairment.<br>*FFB defined as both intermetatarsal and plantar (adventitious) bursa.*
Cherry et al. 2014 [24]	They developed at score-based tool based on fluid (count, shape, enhancement, T1 and T2) and soft tissue (count, shape, enhancement, T1 and T2). Both intermetatarsal and plantar (adventitious) bursas is included. The score showed moderate to good intra- and inter-reader agreement.
Dakkak et al. 2020 [25]	IMB was found in 109 (69%) of RA pts, in one or more interspaces, in 84 (30%) of pts with other arthritis and 31 (16%) of healthy controls.
Dakkak et al. 2020 [26]	516 cases of IMB were found with MRI. It is not stated how many pts this equals. IMB alone explained 21% of the swollen joints found at physical examination.
Dijk et al. 2021 [27]	131 (23%) of pts had IMB on MRI. Their analysis shows IMB as an independent predictor of development of clinical arthritis, particularly in ACPA-positive pts.
Dijk et al. 2021 [28]	IMB behaves in line with known RA characteristics incl. RAMIS-inflammation, treatment response and contributes to typical clinical signs. Supporting that IMB is a juxta-articular involvement in early RA.
Dijk et al. 2022 [29]	IMB contributes to a positive MTP squeeze test in pts with early arthritis.
Endo et al. 2018 [30]	US assessment confirmed IMB with Power Doppler signal in both 2nd and 3rd intermetatarsal space and mild synovitis in one MTP joint on each foot. The clinical finding of opening toes was present between the 2nd and 3rd, and 3rd and 4th toes.
Hammer et al. 2019 [31]	20.6% (43) of pts had IMB either uni- or bilaterally. IMB was most frequent in 3rd interspace (56.5%) followed by the 2nd (33.3%), 4th (5.8%) and 1st (4.3%). IMB was associated with MTP synovitis at US and presence of anti-CCP and RF.
Hooper et al. 2012 [6]	At 3-years follow-up 60 pt. returned. Activity limitations and forefoot impairment associated with the presence of FFB was approaching significant. The authors conclude that FFB are indicative of disability and should be considered for targeted therapy in RA pts.<br>*FFB defined as both intermetatarsal and plantar (adventitious) bursa.*
Hooper et al. 2014 [32]	The prevalence of FFB (defined as ≥ 1 bursa present on US) was 88% in RA pts, 94% in osteoarthritis pts and 56% in healthy controls. A significant difference in distribution of hypertrophied bursas was found between the groups. *FFB defined as both intermetatarsal bursas and plantar (adventitious) bursas.*
Koski 1998 [33]	At US, 14 cases of IMB were found in eight pts (32%). Two healthy controls had US findings suggestive of bursa formation (6.6%). IMB in pts were larger and tender compared to the controls. Due to the spreading of the digits, presence of IMB was clinically suspected in five pts.
Mukherjee et al. 2016 [7]	All pts had minimum one hypertrophied bursa with power Doppler signal at US examination. No significant correlation between US detectable pathology including bursa prevalence and patient-reported foot impairment or activity limitation was found. *FFB defined as both intermetatarsal and plantar (adventitial) bursas.*

IMB: intermetatarsal bursitis, MN: Morton’s neuroma, pt(s): patient(s), MRI: magnetic resonance imaging, US: ultrasound, FFB: forefoot bursas, RA: rheumatoid arthritis, C: healthy controls, SMB: Submetatarsal bursitis

Supplementary Table S3. Overall risk of bias assessed with QUADAS-2.

	Risk of bias				Concerns regarding applicability		
	Patient selection	Index test(s)	Reference standard	Flow and timing	Patient selection	Index test(s)	Reference standard
**Anatomic**							
Bossley et al. 1980 [1]	?	?	?	?	L	L	?
Chauveaux et al. 1987 [5]	L	L	?	L	L	L	?
Theumann et al. 2001 [15]	?	L	L	L	L	L	L
**Metatarsalgia**							
Albano et al. 2021 [16]	L	L	?	L	L	L	?
Awerbuch et al. 1982 [17]	?	?	L	L	L	L	L
Bencardino et al. 2000 [18]	L	H	?	L	L	L	?
Bossley et al. 1980 [1]	?	?	?	?	L	L	?
Cohen et al. 2016 [11]	L	L	L	L	L	L	L
Espinosa et al. 2010 [10]	L	L	?	L	L	L	?
Hassouna et al. 2007 [14]	?	H	?	?	L	L	?
Iagnocco et al. 2001 [13]	?	?	?	?	L	L	?
Umans et al. 2014 [19]	L	L	?	?	L	L	?
Volpe et al. 1998 [12]	H	?	L	?	L	L	L
Zanetti et al. 1997 [20]	L	L	?	L	L	L	?
**Autoimmune disorders**							
Albtoush et al. 2019 [21]	H	H	?	L	L	L	?
Bowen et al. 2010 [22]	L	L	?	L	L	H	?
Bowen et al. 2010 [23]	L	L	?	L	L	H	?
Cherry et al. 2014 [24]	L	L	?	L	L	L	?
Dakkak et al. 2020 [25]	L	L	?	L	L	L	?
Dakkak et al. 2020 [26]	L	L	?	L	L	L	?
Dijk et al. 2021 [27]	L	L	?	L	L	L	?

Dijk et al. 2021 [28]	L	L	?	L	L	L	?
Dijk et al. 2022 [29]	L	L	?	L	L	L	?
Endo et al. 2018 [30]	H	L	?	L	L	L	?
Hammer et al. 2019 [31]	?	?	?	L	L	L	?
Hooper et al. 2012 [6]	L	L	?	L	L	H	?
Hooper et al. 2014 [32]	L	?	?	L	L	H	?
Koski 1997 [33]	?	H	?	?	L	L	?
Mukherjee et al. 2016 [7]	L	L	?	L	L	H	?
